# Multifunctional Conductive Nanofibers for Self‐Powered Glucose Biosensors

**DOI:** 10.1002/advs.202416320

**Published:** 2025-02-18

**Authors:** Seda Gungordu Er, Rameesh Bulathsinghala, Selvinaz Burcu Kizilates, Bing Li, Rucchi Ryan, Tanveer A. Tabish, Ishara Dharmasena, Mohan Edirisinghe

**Affiliations:** ^1^ Department of Mechanical Engineering University College London London WC1E 7JE UK; ^2^ Wolfson School of Mechanical Electrical and Manufacturing Engineering Loughborough University Loughborough LE11 3TU UK; ^3^ The Institute for Materials Discovery University College London London WC1E 7JE UK; ^4^ Advanced Technology Institute University of Surrey Guildford Surrey GU2 7XH UK; ^5^ Radcliffe Department of Medicine University of Oxford Old Road Oxford OX3 7BN UK

**Keywords:** core‐sheath fibers, electroconductive fibers, electrospun nanofiber, graphene oxide, self‐powered biosensor, triboelectric nanogenerator

## Abstract

Electrochemical glucose biosensors are essential for diabetes management, and self‐powered systems present an eco‐friendly and innovative alternative. Traditional biosensors face several limitations including limited sensitivity, enzyme instability, and dependency on external power sources. Addressing these issues, the study develops a novel multifunctional nanofiber integrating biosensor for glucose detection and a self‐powered motion sensor, utilizing an innovative triboelectric nanogenerator (TENG) system. Electrospun nanofibers, composed of graphene oxide (GO), porous graphene (PG), graphene foam (GF), polypyrrole (PPy), and polycaprolactone (PCL), demonstrate enhanced electrical conductivity, triboelectric efficiency, and mechanical strength. Among these, dip‐coated nanofibers exhibited the highest conductivity of 4.9 × 10⁻⁵ S/cm, attributed to superior surface electrical properties of GO. PCL/PPy/GO nanofibers achieved the highest glucose detection performance in cyclic voltammetry and differential pulse voltammetry due to efficient electron transfer mechanisms of GO and PPy. Additionally, triboelectric tests revealed peak voltages of 63V with PCL/PPy/GO and polyvinylidene fluoride nanofibers containing glucose oxidase enzyme. Core‐sheath and dip‐coated nanofibers also demonstrated significant mechanical resilience (∼0.9 N force, ∼350 s durability). These findings highlight PCL/PPy/GO nanofibers as a multifunctional, efficient, and scalable solution, offering highly sensitive glucose detection and non‐invasive sweat analysis along with robust energy harvesting for environmentally friendly and advanced diabetes management systems.

## Graphical abstract

This work develops a novel self‐powered glucose biosensor using polycaprolactone/polypyrrole/graphene oxide (PCL/PPy/GO) nanofibers integrated with a triboelectric nanogenerator. The system achieves high glucose detection sensitivity and generates 63 V from motion, combining robust mechanical strength with eco‐friendly energy harvesting. This multifunctional approach of PCL/PPy/GO nanofibers offers scalable solutions for continuous diabetes management and health monitoring.

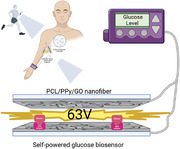

## 1 Introduction

Diabetes is a serious condition that necessitates the regular monitoring of blood glucose levels.^[^ ^1^ ^]^ About 536.6 million individuals worldwide, both diagnosed and undiagnosed, are living with diabetes, and this number is projected to reach 783.2 million by 2045.^[^ ^2^ ^]^ Given that elevated blood glucose levels require immediate medical intervention, the development of biosensors for early diagnosis and continuous monitoring is of critical importance.^[^ ^3^, ^4^ ^]^ Currently, glucose biosensors constitute a significant portion of the biosensor market.^[^ ^5^ ^]^ These sensors are found in different sensing mechanisms, for example colorimetric^[^ ^6^ ^]^ electrochemical,^[^ ^4^ ^]^ fluorescence‐based,^[^ ^7^ ^]^ and optical.^[^ ^8^ ^]^ However, electrochemical glucose biosensors are preferred due to their sensitivity, selectivity, ease of production, and cost‐effectiveness.^[^ ^9^ ^]^ In self‐powered biosensing systems, triboelectric nanogenerator (TENG), which convert the mechanical energy from human body movements into voltage, offer a highly energy‐efficient solution by eliminating the need for an external power supply.^[^ ^10^, ^11^, ^12^, ^13^ ^]^ By incorporating functionalized nanofibers, biosensors can improve both the selectivity and sensitivity of glucose detection, mitigating the impact of interfering substances.^[^ ^14^ ^]^ Additionally, functionalized nanofibers enable the integration of advanced biorecognition elements, providing a robust platform for achieving reliability in glucose sensing applications.^[^ ^15^ ^]^ Nanofibers, due to their high surface‐to‐volume ratio and customizable surface chemistry, can act as selective barriers or matrices that enhance glucose oxidation while reducing the oxidation of other biological species.^[^ ^16^ ^]^ This helps in minimizing false readings and improving the reliability of the performance of the biosensor in complex biological environments.^[^ ^17^, ^18^ ^]^ The analytical performance of enzymatic biosensors can be significantly improved by utilizing nanostructured materials like nanofibers, which offer enhanced surface area, electron transfer, and catalytic efficiency.^[^ ^19^ ^]^

Considering different approaches to develop nanofiber‐based conductive materials, the integration of graphene and its derivatives,^[^ ^20^ ^]^ such as graphene oxide (GO), porous graphene (PG),^[^ ^21^ ^]^ graphene foam (GF)^[^ ^22^ ^]^ and graphene quantum dots (GQDs),^[^ ^23^ ^]^ is a key strategic approach which can facilitate high electrical conductivity, biocompatibility, and ease of functionalization.^[^ ^24^, ^25^, ^26^, ^27^ ^]^ Conductivity of graphene arises from its continuous 2D electron system of delocalized π electrons within its hexagonal lattice, enabling nearly frictionless high‐speed electron movement and an absence of an energy band gap.^[^ ^28^ ^]^ Graphene derivatives also provide a versatile platform for immobilizing biorecognition elements, such as enzymes, antibodies, or DNA probes.^[^ ^29^ ^]^ In addition, electroconductive polymers, such as polypyrrole (PPy),^[^ ^30^ ^]^ polyaniline (PANI),^[^ ^31^ ^]^ poly(3,4‐ethylenedioxythiophene) (PEDOT)^[^ ^32^ ^]^ and polyacetylene (PA), have been demonstrated to produce nanofiber‐based electrically conductive materials. Particularly PPy, a conducting polymer,^[^ ^33^ ^]^ is known for its excellent electrical conductivity due to its highly conjugated π‐electron system. PPy based nanofibers have demonstrated unique advantages such as compatibility with biomolecules.^[^ ^16^ ^]^ Graphene and PPy serve as effective conductive fillers, promoting efficient charge transport within the nanofiber matrix.^[^ ^16^, ^30^, ^34^ ^]^ In addition to these conductive components, other materials such as polycaprolactone (PCL) bring inherent flexibility, biocompatibility, and robust mechanical properties, which enable it to maintain structural integrity under stress, making nanofibers ideal for supporting the wearable and flexible nature of the biosensing devices.

Focussing on fabrication techniques, one of the key challenges in nanofiber forming is achieving uniformity and precise control over fiber morphology. To address these challenges, electrospinning emerges as a versatile and widely used method for producing ultrafine fibers typically ranging from a few nanometers to a few micrometers in diameter.^[^ ^35^ ^]^ This process involves the use of an electric field to draw charged threads of polymer solutions or melts from the tip of a needle toward a collector.^[^ ^36^, ^37^ ^]^ Electrospinning process facilitates a range of exceptional properties for nanofibers such as high surface area‐to‐volume ratio, mechanical strength, and tuneable porosity.^[^ ^38^, ^39^ ^]^ Imparting conductivity into nanofibers via functionalization can be achieved through a diverse range of techniques.^[^ ^40^, ^41^ ^]^ Firstly, conductive materials such as graphene derivatives and PPy can be directly incorporated into the electrospinning solution to fabricate the nanofibers where the conductive materials are mixed throughout the fiber structure. Secondly, a core‐sheath structure can be created where either the inner or the outer layer is conductive. The third is to fabricate the fibers and use dip coating techniques to coat them with conductive materials.

While many studies have investigated the biomedical applications of graphene‐based nanofibers, our research represents a significant advancement in the field by introducing a novel approach that explores the electrochemical glucose measurement potential of electrospun nanofibers composed of PCL, PPy, and graphene derivatives. In this study, various graphene derivatives—including PG, GO, and GF along with PPy were incorporated into PCL nanofibers using distinct methodologies, such as solution direct mix, core‐sheath structuring, and dip coating. These techniques were specifically implemented to enhance the nanofibers’ electroconductivity, ensuring improved performance in biosensing applications. Comprehensive analyses were performed to compare these functionalization techniques in terms of their mechanical properties, biosensing capabilities, and triboelectric performance. Furthermore, a TENG device, embedded with graphene‐loaded nanofibers demonstrated the ability to detect varying glucose levels using the energy it generated. This innovative approach offers a sustainable, self‐powered, and cost‐effective solution for glucose monitoring. A key future goal of our work is to develop a self‐powered biosensor that converts the mechanical energy from arm movements into electrical energy for real‐time, continuous glucose monitoring in sweat, enhancing diabetic patient autonomy and reducing environmental impact.

## 2 Experimental Section

### 2.1 Fabrication and Testing of Multifunctional Nanofiber

**Figure 1:**
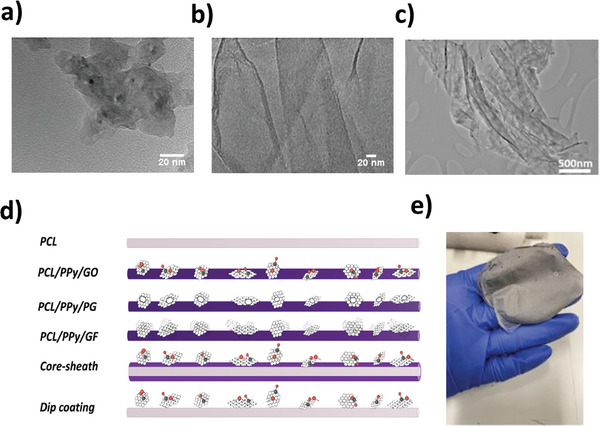
Schematic and TEM Images of Multifunctional Graphene Derivatives‐Based Nanofibers a) TEM image of PG nanomaterial b) TEM image of GO nanomaterial c) TEM image of GF nanomaterial d) Schematic depicting various loading methods of graphene‐based materials and PPy polymer, showing light purple PCL nanofibers and a dark purple mixture of PCL, PPy, and graphene‐based materials. The differences such as functional groups, porosity, and 3D structure among graphene‐based materials (PG, GO, GF) are illustrated e) Actual image of the electrospun PCL/PPy/GO mixed nanofibers produced in the laboratory.

The materials used for the polymer fiber solution were PCL (Mw 80 000 g mol^−1^), PPy, GO PG, and GF (**Figure** **1**a–c). GO (average number of layers 15–20), PG (pore size ≈3–5 nm), and GF (sized ≈4 µm with folded area and number of layers varied from 2–3 to 9–15) were synthesized as reported by Tabish et al. in the previous research.^[^ ^21^, ^22^, ^42^ ^]^ Acetone and distilled water were used as a solvent and the materials were purchased from Sigma Aldrich (Gillingham, UK).

Four different functionalization techniques including pure PCL solution, solution mix, core sheath, and dip coating were chosen for modifying unmodified screen‐printed electrodes (SPEs) and preparing TENG device (Figure 1d). To prepare the pure PCL solution, 8w/v% PCL pellets were kept in acetone under magnetic stir at ≈50 °C overnight. In the mix solution, 8w/v% PCL pellets were dissolved in acetone in a similar way, and 8 w/w% PPy and 4 w/w% GO were added to it and homogenized in an ultrasonic bath for 90 min. It was then left under magnetic stir overnight. The same procedure was followed for PG and GF solution mixtures as for GO. For core‐sheath solutions, the core solution was prepared with the same method as pure 8 w/v% PCL, while the sheath solution was prepared in the same proportions and method as the solution mixed with GO. For the dip coating solution, pure 8w/v% PCL solution was prepared and 4 w/v% GO nanosheets suspension was kept in distilled water in an ultrasonic bath for 90 min.

For nanofiber production, functionalization methods were used by applying electrospinning. To obtain pure PCL, solution mix and dip coated fibers, single needle 18G (1.25 mm BD micro lance) and the capillary tube was polytetrafluoroethylene (PTFE) (outer diameter 2 mm and inner diameter 1 mm). In addition, a dual core‐sheath needle was used in core 18G (1.25 mm BD micro lance) and sheath 14G (2.0 mm BD micro lance) with the same size capillary tube. During electrospinning of all functionalization, the distance between the needle tip and the collector was 150 mm. The humidity in the laboratory was recorded as 43–48%, and the temperature measured in the range of 19–23 °C. For the production of pure PCL nanofibers, the power supply was optimized at 18 kV, and the flow rate was set to 0.1 mL min^−1^. These nanofibers were also used for dip coating. For optimization of the solution mix, the power supply was set to 25 kV, and a flow rate of 0.3 ml/min was used. In the production of core‐sheath nanofibers, the flow rate was optimized to 0.1 mL min^−1^ for the core and the sheath. The power supply was set at 25 kV. Lastly, dip coating technique was conducted by adding GO nanomaterials to distilled water were kept in an ultra‐sonification bath for 90 minutes. Then, the pure PCL nanofibers produced were immersed in GO suspension and kept for 1 min. Subsequently, they were placed on a petri dish and kept in an oven overnight at ≈40 °C to dry.

Viscosity and surface tension tests were conducted on these solutions to evaluate their fluid dynamics and interfacial behavior. These tests are particularly crucial for electrospinning applications, as the viscosity and surface tension significantly influence the formation of nanofibers. The viscosity test was conducted using a Brookfield Viscometer DV‐III (Brookfield, Middleboro, MA, USA). 6 mL of the solution was used for each measurement, and the findings from a small sample spindle were repeated three times and recorded. These measurements were made at room temperature (23 °C).

The surface tension of the solutions was measured using the Du Nouy ring method and a tensiometer (Tensiometer K9, Kruss GmbH, Hamburg, Germany). A 60 mm platinum‐iridium ring was dipped in each solution individually after 10 mL of the solution was placed into a glass bottle. Subsequently, the device was adjusted to maximum hold while being reset to zero, and a nearly detached ring‐liquid meniscus was created. At room temperature, the outcomes were repeated three times, and averages were noted.

Additionally, scanning electron microscopy (SEM) characterization of these nanofibers was performed to analyze their morphology, diameter, and surface structure. The electrospun fibers were placed on aluminum stubs to provide a conductive substrate and reduce charging. The morphology and surface properties were studied using a SEM (Hitachi S‐3400n, Tokyo, Japan) with an operating voltage of 1 kV. To calculate the distribution of fiber diameters, 100 fiber samples were randomly selected from each SEM image and measured using Image J software. The mean and standard deviation of the measured fibers were then calculated and recorded in an Excel file. Finally, the results were graphically represented using OriginPro software. Fourier transform infrared spectroscopy (FTIR) was used to characterize pure PCL, solution mix, core sheath, and dip‐coated fibers. This characterization was carried out using a wavelength range of 3500–500 cm^−1^ and Thermo Scientific Nicolet iS50 FTIR Spectrometer. Kulicke and Soffa model 3007 4‐point prober coupled to a Kiethley 2400 setup was used for sheet resistance measurements. All measurements were done under ambient conditions (45% relative humidity, 24 °C temperature).

Furthermore, tensile tests were conducted on the nanofibers to evaluate their mechanical properties, such as tensile strength and elongation at break. The nanofiber samples for the tensile tests were prepared by cutting them into specimen shapes (5mm width and 4 cm length). The tests were conducted using an INSTRON Tensile Tester with a 5N load cell. Each nanofiber specimen was mounted onto the clamps of the tensile tester and preloaded with a 0.01 N force during the calibration stage. Consequently, the clamps were moved at a rate of 5mm min^−1^ up to the point of their breakage, and the corresponding tensile load values were recorded.

### 2.2 Biosensor Fabrication and Testing

For the biosensor design, SPEs with carbon working electrodes were purchased from Metrohm (DS 110). Flexible conductive silver paste for screen printing, β‐D‐Glucose pentaacetate and glucose oxidase (GOx) (from Aspergillus niger), KCl, K_3_Fe(CN)_6_, K_4_Fe(CN)_6_·3H_2_O^[^ ^31^ ^]^ and all analytes (sodium chloride (NaCl), lactic acid, uric acid, urea,) used in this study for selectivity tests were purchased from Sigma‐Aldrich (Gilingham, UK).

In the preparation of the biosensor, comparative data was obtained by going through certain stages of the obtained graphene‐functionalized nanofibers. For SPEs modification, fiber samples were cut with a sharp circle tool (4 mm diameter) and placed on the working electrode. To ensure that the fibers adhered to the working electrode without decreasing their conductivity, thin silver paste was applied to the working electrode before placing the fibers.

To prepare GOx solution, firstly a pH 7.4 Phosphate buffered saline (PBS) tablet was added to 200 ml of distilled water and stirred overnight on a magnetic stirrer at room temperature. Afterward, 0.2 w/v% GOx was added to the PBS solution and stored in the refrigerator overnight. Then, 20 µL of the GOx solution was immobilized by dripping on each modification using an adjustable pipette and then dried at room temperature (24 °C). The prepared samples were refrigerated overnight.

First, the electrochemical behavior and performance of six different fiber samples (PCL, PCL/PPy/GO, PCL/PPy/PG, PCL/PPy/GF, core‐sheath, dip coating) on SPEs were characterized by cyclic voltammetry (CV) and differential pulse voltammetry (DPV) in 10 mM glucose concentration. Then, PCL/PPy/GO nanofiber was chosen to conduct with 10, 8, 6, 2, and 0.1 mM glucose concentration, because it showed the highest peak in the electrochemical testing. Afterward, electrochemical impedance spectroscopy (EIS) was conducted for all concentrations. CV was conducted at a scanning rate of 100 mV s^−1^ within a potential range of −0.6–+0.6 V. DPV was conducted at a scan rate of 50 mV s^−1^ within a potential range of −0.2–+1.0 V. EIS measurements were carried out under open‐circuit voltage, in the frequency range of 0.1 Hz–100 kHz, and at a signal amplitude of 5 mV. For the selectivity tests, the PCL/PPy/GO nanofiber was evaluated using CV analyses, which were performed three times to ensure consistent results. Electrochemical measurements were conducted using the same procedure adapted from previous work^[^ ^43^ ^]^ 20 mL of 0.01 M PBS solution containing 1 mM K_3_Fe(CN)_6_/K_4_Fe(CN)_6_ and 0.1 M KCl. The concentration of each analyte, including NaCl, lactic acid, uric acid, urea, and glucose, was kept at 10 mM for all tests. This uniformity allowed for a fair comparison of the biosensor response current to different substances under the same conditions. The data from these tests were then compiled into a histogram graph, clearly showing how the biosensor reacted differently to each analyte, demonstrating its selectivity and effectiveness.

### 2.3 TENG Device Fabrication and Testing

#### 2.3.1 Device Design and Fabrication

The TENG fabrication and characterization followed a systematic process to identify the best material combinations, to create an all‐fiber TENG with enhanced output performance.

Herein, the first phase focused on identifying the type of nanofibers with the best triboelectric outputs to be used in the TENG. To achieve this, the nanofiber layers were tested against a reference triboelectric surface, PTFE, which is a commonly used triboelectric material with strong negative charging characteristics. A PTFE film (5 cm × 5 cm × 0.05 cm) was used as TENG layer 1, with a copper film (5cm × 5 cm, 0.09 mm) on the opposite side as the electrode. Each nanofiber film (PCL, PCL/PPy/GO, PCL/PPy/PG, PCL/PPy/GF, core‐sheath, and dip‐coating) with an identical area of 5 cm × 5 cm and a copper film electrode (5 cm × 5 cm, 0.09 mm) on the reverse side, was tested as the counter triboelectric layer (TENG layer 2).

The electrical performance of these TENG configurations was evaluated using a vertical contact‐separation TENG (VCSTENG) architecture, where open‐circuit voltage (*V_oc_*), short‐circuit charge (*Q_sc_*), and short‐circuit current (*I_sc_*) were recorded. Consequently, the material that provided the highest electrical performance (which indicates the most positive material compared to PTFE) was selected as the positive triboelectric layer (TENG layer 2).

The second phase was to replace the PTFE with a nanofiber material, to create the all‐fiber TENG device and to improve the electrical outputs. To this end, Polyvinylidene fluoride (PVDF), located towards the negative end of the triboelectric series, was selected due to its good triboelectric properties as well as the ability to be formed into nanofiber meshes through electrospinning. To produce this layer, Dimethylacetamide (DMAc) and PVDF were obtained from Sigma Aldrich (Gillingham, UK), and a binary solvent mixture of DMAc and Acetone (3:7) was used to prepare a 10 w/v% PVDF nanofiber solution for the TENG device.

The PVDF mixture was stirred overnight using a magnetic mixer to ensure homogeneity. For optimal PVDF nanofiber production, electrospinning was carried out with a flow rate of 0.1 mL min^−1^ and an 18 kV power supply. A 5 cm × 5 cm PVDF nanofiber layer was then prepared as the negative triboelectric layer (TENG layer 1) with a copper film electrode (5cm × 5 cm, 0.09 mm) on the opposite side. This layer was combined with the selected positive triboelectric layer (TENG layer 2) from the first phase, to form the final all‐fiber TENG device, and its electrical performance (*V_oc_*, *Q_sc_*, *I_sc_* and power output) was evaluated.

In the third phase, the compatibility of this device as a multifunctional biosensor was evaluated, where the impact of the presence of GOx and glucose (which relate to its biosensing operation) on the triboelectric performance was assessed. Herein, the nanofibers were first functionalized by immobilizing GOx enzyme onto the nanofiber surface. The immobilization process was carried out overnight at 4 °C to ensure stable enzyme attachment. Following immobilization, the nanofibers were exposed to glucose solutions with concentrations of 5 and 10 mM to simulate varying glucose levels. The triboelectric outputs (*V_oc_*, *Q_sc_*, and *I_sc_*) were measured for each glucose concentration to monitor their variations.

#### 2.3.2 Electrical Characterization

The electrical performance of the TENG device was characterized using a method consistent with our prior studies.^[^ ^44^, ^45^, ^46^ ^]^ Herein, the TENG layers were subjected to contact‐separation movements via a computer‐controlled linear motion system, where a sinusoidal movement with 1 mm amplitude, 1 Hz frequency, and maximum contact force of 10 N was used to simulate typical human movements. The selection of 10 N contact force was based on our previous research^[^ ^46^, ^47^, ^48^ ^]^ and studies from other groups,^[^ ^49^, ^50^ ^]^ to represent a typical force during human motion in wearable TENG devices. To evaluate the electrical outputs, *V_oc_*, *I_sc_* and *Q_sc_*, output power under different loads were characterised using a Keithley 6514 electrometer

These measurements were conducted with the maximum separation between the TENG layers being considered as the baseline (zero potential). During output analysis, the maximum *Q_sc_* and *V_oc_* during each motion, cycle was recorded and averaged (for 20 cycles) to obtain the peak *Q_sc_* and peak *V_oc_* values. For *I_sc_* outputs, there was a difference between the positive and negative current half‐cycles, which can be attributed to the adhesion induced impulsive separation of TENG surfaces.^[^ ^51^ ^]^ Our previous studies^[^ ^51^ ^]^ demonstrated that in such circumstances, the current peaks corresponding to contact half‐cycle movement (in this case, positive current peaks) more accurately represent the current generation of the TENG. To this end, the maximum *I_sc_* corresponding to the positive current half cycles were obtained, and averaged for 20 cycles, to calculate the peak *I_sc_*. Similar procedure was adopted for the power output assessments, where the current outputs through different loads were measured and the peak power (average maximum value for 20 peaks), and mean power (average mean value for 20 peaks) were assessed. The triboelectric tests were conducted under controlled conditions to evaluate the electrical performance, durability, and biosensing compatibility of the TENG device.

## 3 Results and Discussion

Various materials, including PCL, PPy, and graphene derivatives (GO, PG, and GF), were synthesized and incorporated into different configurations, such as pure PCL fibers, mixed PCL/PPy/graphene derivatives, core‐sheath structures, and dip‐coated fibers. The electrospinning process was optimized by adjusting parameters like voltage, flow rate, and needle‐to‐collector distance to ensure the successful production of nanofibers with the desired properties. Following the fabrication, solution testing was conducted, where viscosity and surface tension measurements were taken to refine the solution properties and ensure the formation of fibers with appropriate morphology for further applications.

Once the fibers were prepared, biosensor testing was performed using CV and DPV to evaluate the electrochemical response of each nanofiber configuration in glucose detection. After identifying the best‐performing nanofiber, PCL/PPy/GO, it was further tested across various glucose concentrations (0.1, 2, 6, 8, and 10 mM) to generate calibration curves that demonstrated its sensitivity and precision in glucose detection.

In the next phase, the triboelectric properties of the six different fiber configurations were tested using TENG setup, where Teflon was used as the second layer. The best‐performing fiber was then further examined with both Teflon and PVDF layers, and its triboelectric output, including charge and voltage generation, was thoroughly analyzed. Finally, the selected fiber was functionalized with GOx enzyme, and its performance was tested with glucose concentrations of 5 and 10 mM. This final enzyme‐based testing provided valuable insights into the biosensing capabilities of nanofibers, particularly in response to glucose levels, highlighting its potential for real‐time glucose monitoring applications.

### 3.1 Nanofiber Fabrication and Characterization

**Figure 2:**
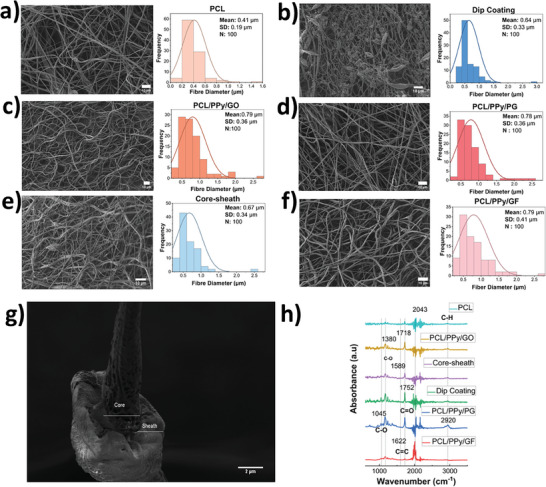
SEM images, fiber distribution graphs, and FTIR results of the six different multifunctional nanofibers a) Pure PCL nanofibers b) Dip coated nanofibers c) PCL/PPy/GO nanofibers d) PCL/PPy/PG nanofibers e) Core‐sheath nanofibers f) PCL/PPy/GF nanofibers g) Core‐sheath SEM image of fiber h) Comparative FTIR results of the multifunctional nanofibers.

PCL nanofibers and PPy, graphene derivatives added fibers were prepared with different functionalizations. SEM results were evaluated to examine the morphology of these nanofibers in more detail. Additionally, fiber diameter distribution histograms were drawn to obtain general information about the diameters. Overall, it is seen that electrospun fibers are nano‐sized and have a beaded structure. The suspensions of both PPy and graphene derivatives affect the entanglement of the fibers together with the power supply. PCL nanofibers, without any additives, were observed to have an average diameter of 0.41 µm. Generally, the surface of these nanofibers is porosity, yet their uniformity seems to surpass other samples. Additionally, beaded fibers, potentially formed due to the rapid evaporation of acetone, are depicted in **Figure** **2**a,c displays the SEM characterization of PCL/PPy/GO fibers, which are approximately twice as thick as PCL nanofibers. The thickest fiber diameter observed for PCL nanofibers is about 1.6 µm, while for PCL/PPy/GO fibers, this value is ≈2.5 µm. Similarly, the fiber morphologies and thicknesses of all three graphene derivatives GO, PG, and GF functional fibers appear to be similar on average, with values of 0.79, 0.78, and 0.79 µm, respectively. While the standard deviations of PG and GO fibers seem to be nearly identical, GF fibers are relatively high. This might be attributed to the 3D structure of GF. Additionally, as demonstrated in previous studies, Figure 2f shows that adding GF increases the structural entanglement of the fibers.^[^ ^20^ ^]^

Dip coated and core‐sheath fibers, which were functionalized to allow more of the GO and PPy to be on the surface, resulted in thinner fibers, unlike direct blends. While the average diameter of the fibers was 640 nm for dip coating, the average diameter of the core‐sheath fibers was 670 nm. When compared to PCL only nanofibers and core‐sheath fibers, the sheath thickness can be shown to be ≈260 nm when evaluated by the difference between the two samples. In this SEM characterization, Figure 2g shows the core‐sheath fiber structure of the fiber surface is revealed.

FTIR analysis (Figure 2h) was conducted for each functional PCL, PPy, and graphene derivative fibers, in line with previous studies found in the literature.^[^ ^24^ ^]^ PCL nanofiber peaks were observed at 2920 cm^−1^ (asymmetric CH_2_ stretching) and 1718 cm^−1^ (carbonyl stretching), which can also be seen in all other fiber samples.^[^ ^52^ ^]^ The fiber with added GO showed peaks of carboxyl groups at 1380 and 1045 cm^−1^, consistent with prior studies.^[^ ^53^ ^]^ Similarly, previous studies indicated that the peaks at 1043 cm^−1^ and 1720 cm^−1^ in the fiber with added PG correspond to epoxy and carbon functional groups.^[^ ^20^ ^]^ Peaks at 1622 and 1380 cm^−1^ in the fiber containing the graphene derivative GF, signify the presence of GF.^[^ ^20^ ^]^ Lastly, GO presence in fibers prepared by core‐sheath and dip coating methods is indicated by the existence of GO on the surface and PCL/PPy values with peaks of 1380 and 1718 cm^−1^.

**Figure 3:**
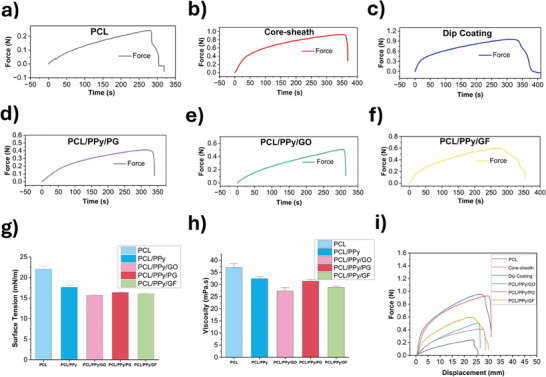
Tensile strength test results of nanofibers and solution viscosity and surface tension a) Pure PCL nanofibers. b) Core‐sheath nanofibers c) Dip coating nanofibers d) PCL/PPy/PG nanofibers e) PCL/PPy/GO nanofibers f) PCL/PPy/GF nanofibers g) Surface tension results of the solutions used for nanofiber fabrication h) Viscosity results of the solutions used for nanofibers i) Comparative force–displacement graph of six nanofibers.

Solutions of PCL, graphene derivatives, and PPy were prepared at determined concentrations. Surface tension and solution viscosity values have an impact on the electrospinning process used to generate the fibers from these solutions. In **Figure** **3**g, the surface tension values of the pure PCL/PPy nanocomposite solutions loaded with PG, GO, and GF are shown. The findings show that compared to the solutions containing PPy and graphene derivatives, the pure PCL solution has comparatively higher surface tension. Additionally, added graphene derivatives slightly reduced the surface tension compared to PPy.

While pure PCL solution showed a surface tension of ≈22 ± 0.8 mN m^−1^ when dissolved in acetone, this value decreases to 17.6 ± 0.5 mN m^−1^ when PPy is added. Similarly, when graphene derivatives were added, this value was measured ≈16 mN m^−1^. GO was found to be the surfactant that had the greatest decreasing impact on the surface tension of the solution it was introduced to. The findings of this research, in conjunction with data from existing literature, indicate that surfactants play a pivotal role in diminishing surface tension.^[^ ^20^, ^54^ ^]^

Similarly, the viscosities of the different solutions examined in Figure 3h were evaluated. It was observed that the PCL solution was relatively more viscous compared to other solutions added with PPy and graphene derivatives. While 8 w/w % PCL solution has a viscosity of ≈37 ± 1.2 mPa.s, PPy and GO added show a viscosity of ≈26 mPa.s. Similarly, GF was measured ≈27 mPa.s, but the other graphene derivative PG affected the viscosity less and showed a value at 31 ± 0.8 mPa.s. Graphene‐based materials in this study act like surfactants, reducing surface tension through electrical forces between particles. The decrease in viscosity may be caused by the instability of the added nanomaterial such as GO, PG, GF, and PPy in the solution.^[^ ^55^ ^]^

In the study, an exploration of the force responses of the manufactured fibers was conducted (Figure 3). The results indicated that nanofibers, created solely with PCL, could endure a peak force of 0.25 Newton (N) prior to breaking, typically within a time frame of 250 to 300 s. Research literature echoes the superior mechanical strengths of graphene and its derivatives. Indeed, fibers that were produced from blended solutions, with the incorporation of graphene derivatives within PCL and the addition of PPy, showcased a higher degree of mechanical resistance when force was applied.

GO demonstrated a resistance to ≈0.5 N of force for a duration of 320 s before breaking (Figure 3e). Comparatively, PG withstood a force of up to 0.4 N for 360 s before a fracture occurred (Figure 3d). The lower force resistance exhibited by PG could potentially be ascribed to its porous structural composition. Among the derivatives, GF proved to be the most resilient, breaking at a force measurement of 0.6 N and at 300 s (Figure 3f).

Fibers produced through the core‐sheath and dip coating techniques displayed a resistance to substantially higher forces, ≈0.9 N (Figure 3b,c). These fibers generally reached their breaking point around the average time of 350 s. As a result, when graphene is incorporated into PCL nanofibers, it acts as a reinforcing filler, increasing the overall stiffness of the composite material. The mechanical stress tests were conducted to evaluate the nanofibers' durability and structural robustness under continuous operation, simulating sustained stress conditions to ensure their reliability in practical applications. Jalaja et al. have documented that integrating GO into polymer matrices significantly enhances their mechanical properties.^[^ ^56^ ^]^ Similarly, Wan et al. reported an increase in tensile strength of gelatin films following the incorporation of GO, underscoring the beneficial effects of GO on the mechanical performance of polymer‐based materials.^[^ ^57^ ^]^ The inherent strength of GO sheets combined with their ability to form cross‐linking networks results in superior tensile and compressive behaviors. This is crucial for the durability of wearable sensors, which require flexibility and robustness.

### 3.2 Biosensor Performance of Nanofibers

Graphene‐based nanocomposites achieve exceptional electrical conductivity, enabling cutting‐edge biomaterials application.^[^ ^16^, ^31^, ^58^, ^59^, ^60^, ^61^ ^]^ Preparing nanofibers can further enhance the mechanical properties of these composites.^[^ ^62^ ^]^ Percolation theory plays a crucial role in understanding the phenomenon between graphene‐based materials and polymer.^[^ ^34^, ^60^, ^63^ ^]^ According to percolation theory, when the concentration and functionalization of graphene‐based materials in a polymer matrix reaches a critical percolation threshold, a continuous conductive network forms, drastically enhancing the electrical conductivity of the composite. This continuous network allows electrons to move freely throughout the material, significantly improving its overall electrical conductivity. In this study, graphene‐based materials and the electrically conductive polymer PPy were used together to ensure the continuity of the connection network. Additionally, an increase in connectivity was expected, especially for graphene located predominantly on the surface.

**Figure 4:**
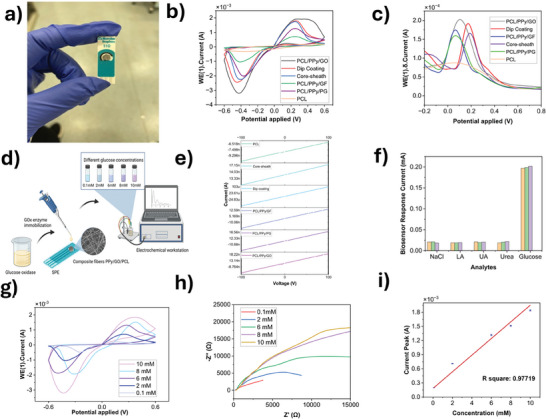
Electrical and electrochemical analysis of the multifunctional fibers, electrochemical biosensing results of PCL/PPy/GO nanofibers a) real image of PCL/PPy/GO nanofiber modified SPEs on the working electrode b) comparative CV results of the nanofibers c) comparative DPV results of the nanofibers d) Schematic of step by step preparation of nanofibers for electrochemical analysis e)Comparative electrical conductivity results of the nanofibers f)Selectivity results of PCL/PPy/GO nanofibers with different analytes (Sodium chloride (NaCl), lactic acid, uric acid, urea, and glucose) g) CV results of PCL/PPy/GO nanofibers for 0.1, 2, 6, 8, and 10 mM concentration glucose h) EIS results of PCL/PPy/GO nanofibers for 0.1, 2, 6, 8, and 10 mM concentration glucose i) Calibration curve for CV current peaks.

The electroconductivity of the obtained nanofibers was evaluated using 4‐point probes (**Figure** **4**e). The samples were cut to the same size for this analysis. In the comparative analysis results, the fibers obtained by the 103 µA dip coating method showed the highest current values while PCL nanofibers showed the lowest value 6.518 nA. The electrical conductivity of a nanofiber can be determined using the following equation:

(1) $$\sigma ={\left[\frac{V}{I}\;.\;\frac{w.t}{S}\right]}^{-1}$$

In this equation, *V* represents the voltage difference, *w* is the width of the nanofiber, *t* is the fiber thickness, *I* is the applied current, and *S* is the electron separation.

Dip coated fibers showed the highest electrical conductivity which was calculated at 4.9 × 10^−5^ S cm^−1^. The primary reason is likely to be because the fiber is completely submerged in the GO suspension during the process. This complete immersion allows the GO to fully adhere to the entire surface of the fiber, contributing significantly to its electroconductivity. In previous work, Santos et al.^[^ ^64^ ^]^ found that the electrical conductivity of the nanofibers they developed varied significantly, ranging from 10^− 13^ S/cm to 10^−1^ S cm^−1^. This variation was attributed to the differences in polymer types and nitrogen‐doped carbon nanotubes (N‐CNTs) utilized. They specifically noted an enhancement in electrical conductivity with the incorporation of PAni within Polyamide 6 (PA6), which facilitated a higher deposition of N‐CNTs on the nanofibers, thereby improving the overall electrical conductivity of the nanostructures. Chronakis et al.^[^ ^30^ ^]^ observed a significant enhancement in the conductivity across the thickness of electrospun PPy/polyethylene oxide (PEO) nanofibers, which escalated by approximately two orders of magnitude correlating with an increase in PPy concentration. The range of conductivity observed spanned from roughly 4.9 × 10^−8^ to 1.2 × 10^−5^ S cm^−1^, indicating the substantial impact of PPy content on the electrical properties of the nanofibers. The outer surface of the core‐sheath fibers and PCL/PPy/GO solutions have similar content, showing values of 17.2 and 18.2 nA, respectively (Figure 4e). The slightly lower value for the core‐sheath might be due to the core being composed entirely of PCL. The electrical conductivity values of GF and PG derivatives added nanofibers were measured with great care; the lower values in comparison to other derivatives exhibited by GF added nanofibers. As the size of GF increases, its electrical conductivity improves. GF with a large flake size over 100 µm shows even better results.^[^ ^60^ ^]^

CV and DPV current response curves have been used to characterize the electrochemical behavior of the modified SPE electrode by 6 different functionalized nanofibers. Figure 4b shows the CV curves of the modified electrode while Figure 4c demonstrates the DPV current of these modified electrodes in 10 mM glucose. These two graphs were applied to examine the comparative CV and DPV results of functional nanofibers where exactly the same processes were applied.

CV and DPV results of PCL nanofibers showed the lowest current peak. It is thought that this is because it does not contain conductive polymers and conductive nanoparticles. The highest current peak was found in the results obtained with PCL/PPy/GO. The reason for the better result may be that GO contains oxygen‐containing functional groups, such as hydroxyl and carboxyl groups. These functional groups facilitate the binding of the GOx enzyme.^[^ ^65^ ^]^ Core‐sheath nanofibers and dip coated nanofibers are showing slightly higher current peaks compared to others. For core‐sheath structure, the outer sheath can maximize the interaction with the target analyte by covering conductive materials mostly on the surface. PCL/PPy/PG and PCL/PPy/GF showed lower current peak results compared to the others. The reason for this is the 3D nature of GF and the porositic structure of PG might have lead to reduced accessibility of target analytes to the sensing surface.

For further CV and EIS analysis, PCL/PPy/GO was used as it displayed the highest CV and DPV peaks at a glucose concentration of 10 mM. The figure includes multiple curves, each representing a different glucose concentration of the test sample, as indicated by the legend (10, 8, 6, 2, and 0.1 mM) (Figure 4g). Notably, the peak current decreases as the glucose concentration decreases. The largest peak currents correspond to the 10 mM glucose concentration, suggesting that the electrode surface modified by PCL/PPy/GO nanofibers is sensitive to glucose concentration. Furthermore, Figure 4h shows the EIS analysis of these glucose concentrations. The semicircle radius (Rct value) increases as the glucose concentration increases.

The calibration curve current peaks were evaluated by three separate experiments (Figure 4i). The calibration curve shows that current peaks increase linearly with the logarithmic concentration of glucose in the range of 0.1−10 mM, with a correlation coefficient of 0.977. Additionally, the limit of detection (LOD) for the biosensor was calculated utilizing 0.21 µM. PCL/PPy/GO nanofibers immobilized with GOx enzyme are shown in the selectivity graph (Figure 4f)along with its response to several analytes (NaCl, lactic acid, uric acid, urea, and glucose) at a concentration of 10 mM. There is a significantly increased current response to glucose, which is roughly 0.20 mA. The reactions to urea, uric acid, lactic acid, and NaCl, on the other hand, are all noticeably reduced below the 0.05 mA threshold. This significant difference indicates less interference from other compounds and highlights the immense selectivity response towards glucose. To summarize, the PCL/PPy/GO nanofibers demonstrate notable sensitivity and selectivity to varying glucose levels, indicating their potential as effective components in glucose biosensors.

In the literature, there are numerous examples of enhanced biosensor properties achieved through the nanocomposite modification of electrodes. Arvand et al.^[^ ^66^ ^]^ confirmed through their research findings that integrating titanium nanofibers (TNFs) into GO nanocomposites significantly enhances electrode efficiency. This improvement is attributed to the presence of TNFs, which, due to their high surface‐to‐volume ratio, increase the number of accessible active sites, as well as facilitate easier electron transfer within the TNFs/GONs nanocomposite. Consequently, TNFs and GONs exhibit a synergistic effect that optimizes the performance observed in CV results. Promphet et al.^[^ ^67^ ^]^ have indicated that the notable augmentation in current response suggests an enhancement in the electrochemical sensitivity of the unmodified screen‐printed carbon electrode due to the incorporation of G/PANI/PS nanoporous fibers. This enhancement hints at the potential utility of their modified electrode in the development of sensitive electrochemical sensors, underlining the significant impact of these nanoporous fibers on sensor performance. Many traditional enzymatic glucose biosensors exhibit LODs in the range of 1−100 µM, depending on the sensing material and immobilization techniques. This study reports a novel electrochemical glucose sensor with two linear response ranges (0–1200 µM and 1200–5000 µM) and LOD of 18.64 µM, showcasing its selective glucose sensing capabilities. Our biosensor platforms show greater sensitivity (LOD: 0.21 µM versus 18.64 µM) and a broader detection range (0.1−10 mM), offering superior precision and versatility.^[^ ^68^ ^]^ Also, the incorporation of GO into PCL nanofibers significantly enhances their mechanical stability, as evidenced by their ability to resist forces up to 0.9 N and maintain structural integrity for an average of 350 s, ensuring robustness under prolonged mechanical stress critical for biosensors.

In the field of functional nanofibers, graphene has been extensively investigated in various biomedical research studies. In this study, PG, GO, and GF were comparatively analyzed for the first time. The findings indicate that the electrochemical properties of GO‐based nanofibers are more significant compared to other derivatives. In electrochemical biosensing, GO facilitates the immobilization of biomolecules because it has oxygenated functional groups, thus increasing sensitivity.^[^ ^69^ ^]^

The incorporation of graphene and conductive polymers onto the surface of nanofibers through methodologies such as dip coating and core‐sheath forming has been shown to be effective in improving both the electrical and mechanical properties of these materials.^[^ ^30^, ^59^, ^70^ ^]^ Core‐sheath nanofibers allow graphene to be strategically aligned on the surface, thus enhancing mechanical strength while maintaining electrical conductivity.^[^ ^71^ ^]^ Such structures are particularly advantageous for wearable devices where flexibility and durability are as critical as electrical performance. These advanced properties both expand the applicability of nanofibers in various fields and pave the way for innovative designs in the field of self‐powered electronics and advanced biosensors.

### 3.3 Self‐Powered Motion Biosensing Performance of Nanofibers

The nanofibers developed in this work contain static charging properties, allowing them to function as self‐powered motion sensors based on the TENG technology. Here, selecting an optimum combination of triboelectric materials becomes a key design aspect. To this end, a material analysis was conducted as described in section 2.3.

**Figure 5:**
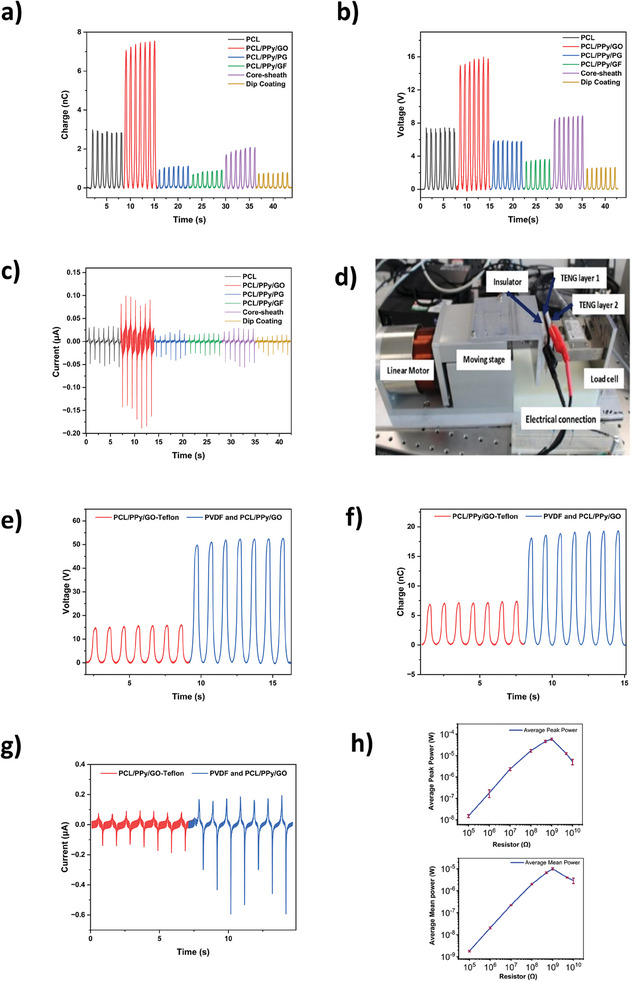
Triboelectric efficiency of the multifunctional fibers and for two layers of TENG device separately and together a) Comparative output charge b) Comparative output voltage results c) Comparative output current results. d) Triboelectric testing of two layers of TENG device. e)Output voltage of two layers f) Output charge over time of two layers. g) Output current over time of two layers h) Experimental average peak output power measured through load resistors (indicated as previous study^[^ ^32^ ^]^) and mean output power measured through load resistors.

In the first phase of the triboelectric study, different nanofiber structures were tested against a reference PTFE sheet to assess their triboelectric performance. The experimental outputs for the *Q_sc_* are shown in **Figure** **5**a, where PCL/PPy/GO nanofiber layer showed the highest performance with peak *Q_sc_* of ≈7.31 nC, followed by 2.85 nC for PCL, 1.91 nC for the core‐sheath nanofiber layer, 1.05 nC for PCL/PPy/PG, 0.81 nC for PCL/PPy/GF, and 0.75nC for the dip coated nanofiber layer. Furthermore, the *V_oc_* outputs for these nanofiber layers are shown in Figure 5b, followed a similar trends to *Q_sc_*, with PCL/PPy/GO showing the highest peak *V_oc_* of 15.48 V, followed by 7.71 V for PCL, 8.75 V for core sheath nanofibers, 5.57 V for PCL/PPy/PG, 3.55 V for PCL/PPy/GF, and 2.58 V for the dip coated nanofiber layer. Moreover, the *I_sc_* outputs (Figure 5c) agreed with this trend, with PCL/PPy/GO showing the highest peak *I_sc_* of 90.01 nA, followed for 30.92 nA by PCL, 26.24 nA for core sheath nanofiber layer, 17.33 nA for PCL/PPy/PG, 16.33 nA for PCL/PPy/GF, and 12.26 nA for the dip coated nanofiber layer. It was evident that the PCL/PPy/GO nanofiber layer provided best electrical outputs against the reference, negative triboelectric surface (PTFE) compared to all other nanofiber layers. Hence, this was selected as the positive triboelectric layer for the final TENG design. The reason behind its higher performance could be the capability of GO composites being efficient at electrostatic induction, contributing to improvements in TENG performance.

The focus of this study was to create an all‐nanofiber TENG design with improved outputs, therefore, the PTFE layer was replaced in the second phase of this study with a triboelectrically negative nanofiber layer. Herein, PVDF, which is known for being a negative triboelectric material and which is compatible with electrospinning, was introduced as the negative triboelectric material. To assess its compatibility and performance, a cross‐comparison of electrical outputs was carried out where the PVDF nanofiber layer was tested against the PTFE sheet as well as the PCL/PPy/GO nanofiber layer.

The *V_oc_*, *Q_sc_* and *I_sc_* outputs related to this analysis are depicted in Figure 5, where the output levels between PTFE and PCL/PPy/GO triboelectric layers are also depicted in the same graphs for comparison purposes. Looking at *V_oc_* outputs (Figure 5e), the TENG design containing PVDF nanofiber layer and PCL/PPy/GO nanofiber layer produced the highest peak *V_oc_* of 51 .92V. A similar trend was observed for *Q_sc_* and *I_sc_*, with the same TENG design providing the highest *Q_sc_* of 18.93 nC and *I_sc_* of 172.45 nA, compared to the other two TENG configurations. Therefore, using the PVDF as the negative triboelectric layer not only facilitated the construction of the all‐fiber TENG, but significantly increased the electrical outputs. These output improvements can be attributed to the increase in effective contact surface area (nanoscale rubbing) introduced by the use of PVDF nanofibers instead of the PTFE sheet.^[^ ^72^ ^]^ Consequently, the peak and mean power outputs of this all‐fiber TENG architecture were assessed experimentally, as depicted in Figure 5h,i respectively. These power outputs follow the characteristic load response behavior of a TENG where the power gradually increases with the increasing load, peaks at the optimum load, and decreases afterward. The maximum peak power is 58.77 µW via a 1GΩ load. Similarly, the maximum mean power is around 1 µW at a matched resistance of 1GΩ.

**Figure 6:**
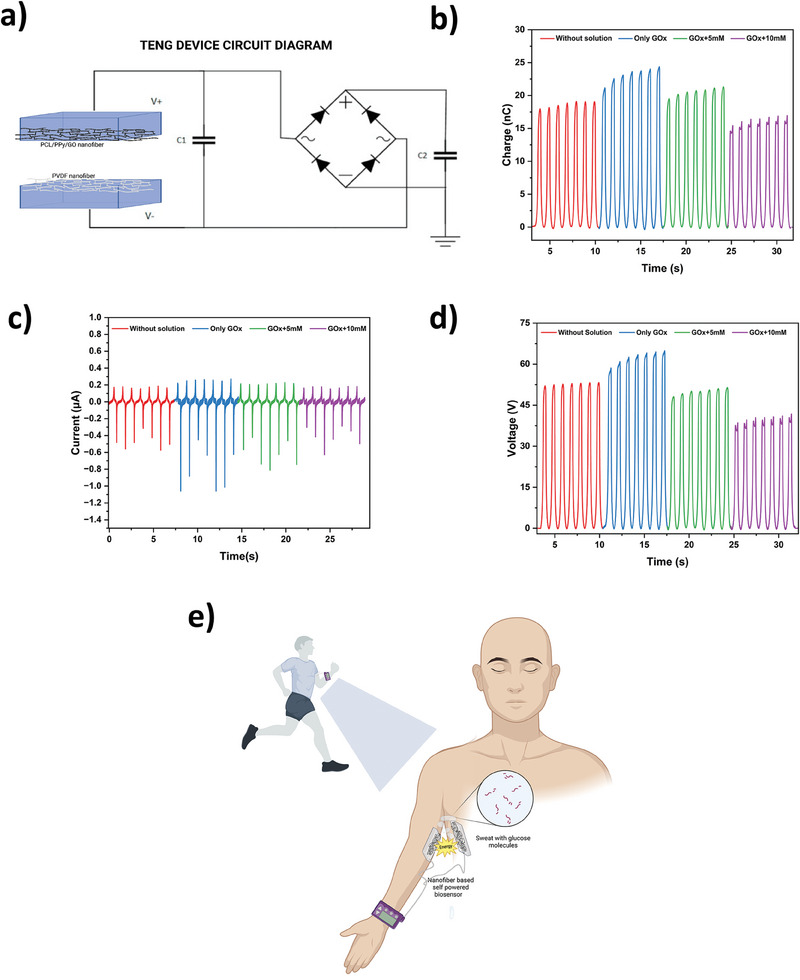
TENG device testing with GOx and different glucose concentration a) TENG Device circuit diagram b) Output charge without GOx, only with GOx, GOx and 5Mm, GOx and 10 mM c) Output current without GOx, only with GOx, GOx and 5Mm, GOx and 10 mM d) Output voltage without GOx, only with GOx, GOx and 5Mm, GOx and 10 mM e)Illustration of next generation nanofiber based self‐powered biosensor to measure glucose levels from the sweat.

From section 3.2, it was evident that the PCL/PPy/GO nanofiber layer showed the highest sensitivity to varying glucose levels, whereas the same layer provided the highest triboelectric performance and became the positive triboelectric layer in the all‐fiber TENG. Therefore, in the third phase of the triboelectric study, the compatibility of this TENG architecture in working as a multifunctional sensor was assessed in **Figure** **6**. Herein, the TENG was tested under three different conditions, with GOx (0.2 w/v%), GOx (0.2 w/v%) + 5 Mm glucose, and GOx (0.2 w/v%) + 10 mM glucose, to observe its electrical output variations. The experimental peak *V_oc_* outputs are shown in Figure 6d, which increased from 51.92 to 62.32 V when GOx was added to the PVDF‐PCL/PPy/GO TENG. However, the *V_oc_* decreased with increasing glucose concentrations, dropping to 49.85 V for GOx + 5 mmol glucose and to 39.57 V for GOx + 10 mM glucose. A similar trend was observed for *Q_sc_*, (Figure 6b) and *I_sc_*, (Figure 6c) where initial *Q_sc_* and *I_sc_* were 18.93 nC and 172.45 nA, respectively, which rose to 23.15 nC and 250.08 nA with the addition of GOx, but then decreased to 20.49 nC and 198.4 nA for GOx + 5 mM glucose solution, and further to 15.96nC and 170.54nA for GOx + 10 mM glucose solution.

Analyzing these output trends, the output increase following the addition of GOx can be attributed to its strong electron‐accepting properties. Due to this, GOx can facilitate the generation of more positive charges on the PCL/PPy/GO nanofiber layer. Higher charges result in increased surface charge densities, which enhances electrical outputs.^[^ ^44^, ^45^, ^46^ ^]^

However, when glucose is added to this surface, the immobilized GOx catalyzes the oxidation of β‐D‐glucose by molecular oxygen, which can produce gluconic acid and hydrogen peroxide.^[^ ^73^, ^74^ ^]^ The hydrogen peroxide releases electrons upon oxidation, leading to an accumulation of negative charges,^[^ ^74^ ^]^ which can contribute to reducing the positive charge density of the PCL/PPy/GO layer.

As the glucose concentration increases, this accumulation of negative charges also rises, further reducing the positive triboelectric charge density of the PCL/PPy/GO nanofiber layer. This, in turn, leads to a further decrease in the TENG electrical outputs. However, it should be noted that even in the presence of GOx and different glucose concentrations, the TENG produced distinct and excellent electrical outputs, proving its capability to work as a self‐powered motion sensor and an energy harvesting device.

Therefore, this work demonstrates the ability of integrating triboelectric and electrochemical biosensing into a single nanofiber type (PCL/PPy/GO), which is a significant advancement towards future self‐powered biosensor applications. Compared to traditional biosensors, our platform offers a more eco‐friendly solution by eliminating the need for disposable batteries and utilizing nanofibers made from biodegradable polymers, addressing both electronic waste and material sustainability challenges. The triboelectric property of these nanofibers allows them to convert mechanical energy into electricity, which opens potential applications in self‐powered motion sensing and energy harvesting.^[^ ^10^, ^57^, ^58^ ^]^ This biosensor has potential applications in wearable health monitoring devices for continuous glucose tracking and self‐powered medical patches. Additionally, its multifunctional capabilities could be integrated into fitness trackers or smart textiles for monitoring both physical activity and biochemical parameters simultaneously. Concurrently, the electrochemical biosensing functionality enables the nanofibers to detect biochemical changes, such as glucose levels, facilitating non‐invasive health monitoring. The combination of these features offers tremendous potential for multifunctional sensors in wearable technology. Further optimization of these nanofibers like refining nanofiber fabrication techniques can lead to significant advancements in remote healthcare, where continuous monitoring of both mechanical and electrochemical parameters is essential.

In the next phase of this study, a next‐generation biosensor that harnesses the mechanical energy from the arm movements of a patient during physical activities such as running is elucidated(Figure 6e). This self‐powered biosensor will be strategically placed under the arm, where it can convert kinetic energy into electrical energy, eliminating the need for external power sources. This advancement will enable continuous, real‐time monitoring of glucose levels in sweat, providing diabetic patients with immediate feedback on their condition. By integrating this technology, we aim to enhance patient autonomy, minimize environmental impact, and improve accessibility to precise health data, paving the way for more personalized and responsive diabetes management.

The use of nanofibers in triboelectric and electrochemical biosensing devices offers many advantages and inherent challenges.^[^ ^75^, ^76^, ^77^, ^78^ ^]^ On the one hand, their high surface area to volume ratio, lightweight structure, and porosity make nanofibers ideal for sensitive biosensing, allowing rapid detection and high loading of biosensitive elements. This same morphology is known to be useful for triboelectric applications where contact surface area is a critical factor for effective energy harvesting.^[^ ^79^ ^]^ However, the complexity of nanofiber production can lead to significant disadvantages.^[^ ^80^ ^]^ The reproducibility of nanofiber properties such as diameter, distribution, and alignment, which are crucial for consistent device performance, can be difficult to control during manufacturing.^[^ ^81^ ^]^ Additionally, the long‐term stability and durability of these nanofibers may be problematic, especially when exposed to harsh environmental conditions or mechanical stress. These challenges reveal the need for advanced manufacturing techniques^[^ ^82^ ^]^ and robust material formulations to realize the full potential of nanofiber‐based devices in practical applications.

## 4 Conclusions

In this study, various functionalized nanofibers were examined through mechanical tests, electrical conductivity assessments, glucose biosensor results using SPEs, and their triboelectric properties. Six different types of nanofibers were tested, including PCL/PPy/GO, PCL/PPy/PG, PCL/PPy/GF in a solution mix, as well as core‐sheath, dip‐coated, and pure PCL fibers. SEM results have shown that the addition of GO and PPy conductive polymers resulted in an increase in fiber diameter. While pure PCL fibers had a diameter of ≈400 nm, the solution mix fibers exhibited diameters ≈800 nm. For the surface‐only configurations such as core‐sheath and dip coating, the fiber diameter was ≈650 nm.

These findings indicate that the incorporation of conductive polymers not only enhances the electrical properties but also affects the structural dimensions of the fibers, which could have implications for their mechanical strength and overall performance in various applications. In the conducted mechanical strength tests and electrical conductivity assessments, the best results were observed when the GO surface coverage was higher, particularly in the core‐sheath and dip‐coated configurations. These configurations displayed superior mechanical resilience and enhanced electrical conductivity, showcasing their potential for robust and efficient applications in sensor technologies.

This correlation highlights the critical role of GO surface enhancement in optimizing the physical and electrical properties of the nanofibers, underscoring the importance of material design in the development of advanced functional materials. The biosensing capabilities, as well as the triboelectric properties of PCL/PPy/GO, were further investigated at different glucose concentrations, demonstrating dose‐dependent results with the highest CV readings ≈0.2 mA. These biosensing features were supported by calibration curves and EIS tests. Simultaneously, when the triboelectric properties were tested, having PCL/PPy/GO as the positive charge and PVDF as the negative charge led to voltage peaks reaching 63 V. This value reached its maximum when GOx was added and began to decrease with the addition of 5 and 10 mM glucose. Conclusively, this study has developed a highly sensitive and selective, cost‐effective, easy‐to‐produce, self‐powered biosensor. It shows promising potential for diabetes management solutions, offering a significant step forward in the development of non‐invasive, continuous monitoring technologies.

## Conflict of Interest

The authors declare no conflict of interest.

## Author Contributions

S.G.E. conceived the entire project and performed conceptualization, resource management, experiments, data analysis, visualization, and wrote the original draft. R.B. was involved in experiments, visualization, data analysis, and wrote the original draft. S.B.K., B.L. and R.R. contributed to the experiments, provided resources, and reviewed and edited the manuscript. T.A.T. provided resources, reviewed and edited the manuscript. I.D. provided resources, was involved in conceptualization, supervision, reviewed and edited the manuscript. M.E. provided resources, was involved in conceptualization and overall supervision, reviewed and edited the manuscript.

## Data Availability

The data that support the findings of this study are available from the corresponding author upon reasonable request.
